# How Many Diseases Are Colorectal Cancer?

**DOI:** 10.1155/2012/564741

**Published:** 2012-09-09

**Authors:** A. Greystoke, S. A. Mullamitha

**Affiliations:** ^1^Department of Medical Oncology, Christie NHS Foundation Trust, Manchester M20 4BX, UK; ^2^School of Cancer and Imaging Sciences, University of Manchester, Manchester M13 9PL, UK

## Abstract

The development of personalised therapy and mechanism-targeted agents in oncology mandates the identification of the patient populations most likely to benefit from therapy. This paper discusses the increasing evidence as to the heterogeneity of the group of diseases called colorectal cancer. Differences in the aetiology and epidemiology of proximal and distal cancers are reflected in different clinical behaviour, histopathology, and molecular characteristics of these tumours. This may impact response both to standard cytotoxic therapies and mechanism-targeted agents. This disease heterogeneity leads to challenges in the design of clinical trials to assess novel therapies in the treatment of “colorectal cancer.”

## 1. Introduction

Incremental improvements in the outcome of patients with metastatic colorectal cancer have been seen over the last 20 years as initially new cytotoxic agents, and more recently agents targeting the biological abnormalities of the cancer (mechanism-targeted agents (MTAs)) are integrated into routine clinical practice [1]. Until recently treatment for metastatic colorectal cancer was mainly guided by host factors such as age and performance status, rather than tumour factors such as anatomical location or molecular profile. The increasing use of MTAs where activity may be restricted to tumours expressing a particular target means that there is increasing interest in the molecular classification of tumours. Improving the classification of CRC may enable better estimation of prognosis and identify the patients most likely to respond to novel targeted agents.

This change in approach from selecting therapy purely on the basis of the primary site of origin to a more stratified approach has perhaps best been exemplified by work done in the treatment of breast cancer [2]. Initial classification of the cancer by hormonal receptor expression, was supplemented by the measurement of the expression of the oncogene Her-2 as a predictive biomarker of response to the trastuzumab. Molecular profiling then led to further classification into basal, luminal A, and B subtypes according to the presumed cell of origin; most recently it has been suggested that there exist at least 10 different molecular subtypes of breast cancer [3], with potentially differential responses to therapies. Similar advances have been made in the molecular classification of other cancers (e.g., nonsmall cell lung cancer, diffuse large B-cell lymphoma [4, 5]) and is being advocated in the treatment of CRC [6]. This obviously has important implications in the design and interpretation of trials of novel therapeutics in these areas. 

The additional effort and expense of tumour classification on molecular grounds may not be useful if similar information can be obtained from standard clinical and histopathological features. Much of the work to date in CRC has examined the prognostic impact of anatomical and histological differences on prognosis, especially in the surgically resected setting. These studies may give us clues as to differences in clinical behaviour and as will be discussed many of the differences in clinical and histological characteristics are associated with different molecular profiles. It is likely that similarly to other diseases, these differences in molecular profiles will eventually lead to personalised therapies that take into account heterogeneity in colorectal cancer. 

This paper will discuss the advances that have been made in anatomical, histological, and molecular classification of CRC and the potential impact on trial design of novel therapeutics.

## 2. Anatomical Classification

The retroperitoneal position of the rectum in the pelvis requires differences in the management of localised rectal cancer compared to colon cancer. Surgery alone for rectal cancer leads to local recurrence rates that are similar to the incidence of distal metastases, particularly if the circumferential margin is involved [7]. The adoption of total mesorectal excision and preoperative chemotherapy for stage 2 and 3 tumours has led to significant reductions in local recurrence and is now standard practice in the management of these cancers [8–10]. Although local recurrence is associated with a poor outcome, the use of chemoradiotherapy has yet to be shown to have a significant effect on overall survival [10]. The differences in initial management of rectal cancers have meant that patients with these tumours have been excluded from trials evaluating the benefit of adjuvant chemotherapy in colon cancer [11]. 

It is not only the presentation and management of localised CRC that varies dependent on the primary site of the cancer. There is a marked change in bowel contents along the length of the large intestine. The exposure of the epithelia to carcinogens (both in terms of the agents and length of exposure) therefore differs from the right side to the left side of the large intestine, and unsurprisingly this results in a different pattern of molecular abnormalities [12]. There is increasing evidence that the epidemiology, carcinogenesis, molecular profile, and clinical behaviour of colorectal cancer may differ depending on where along the length of the large intestine it initially develops. However, in patients with metastatic disease, no distinction is made between the treatment of colon and rectal cancer [13]. Trials such as the MRC Focus trial which evaluated the optimum sequence and combination of chemotherapy enrolled patients with both colon and rectal cancer and found no difference between the two groups [14].

It was initially suggested that cancers are divided according to whether they arose within the embryonic mid-gut and hindgut (approximately at the splenic flexure) due to differences in epidemiology, tumour morphology, and molecular biology between right-sided and left-sided tumours [15]. Whilst this classification is easily applicable both clinically and within trials, it is probably a major over-simplification and a gradual change between the histological and molecular characteristics between tumours arising in the ascending colon and those in the rectum probably occurs, with no discrete transition point [12].

The histological and molecular differences will be described in more detail below, but the differences in aetiology are reflected in observed trends in epidemiology and clinical behaviour. Increasing rates of right-sided bowel cancer have been seen recently in some countries [16–18], thought to be partially related to changes in diet and intestinal microflora; this has been accompanied by a reduction in rectal tumours. Interestingly, the trend of increasing proximal cancers has been reversing in the USA, possibly due to high rates of colonoscopic surveillance [19].

**Figure 1:**
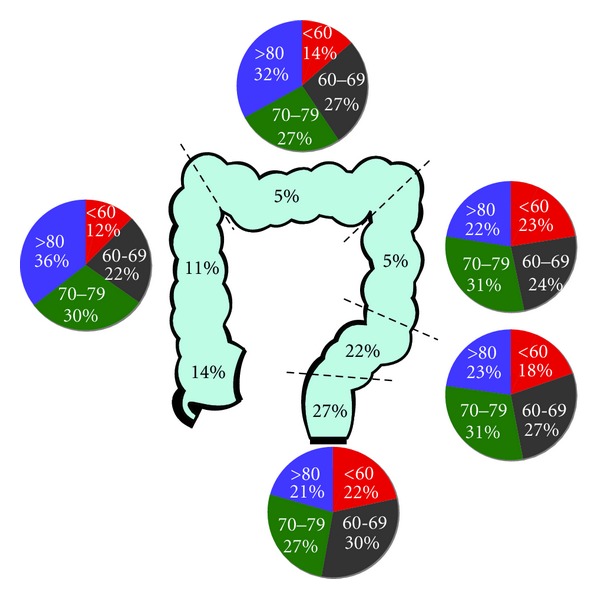
Epidemiology of colorectal cancer by primary tumour site (distribution of tumour sites from UK national statistics (http://info.cancerresearchuk.org/cancerstats/types/bowel/incidence/uk-bowel-cancer-incidence-statistics; age distribution by tumour site from Hemminiki et al. [26]).

It appears that risk factors for the development of proximal cancers differ from the more distal cancers. A higher proportion of proximal cancers are found in women and older patients with CRC [20, 21] (see Figure 1). In addition, other epidemiological factors that are associated with higher risk of proximal cancer have been identified including cholecystectomy which is related to an increased incidence of proximal cancers only [22], whilst obesity is more strongly related to the development of proximal compared to distal cancers [23]. In some series, nonalcoholic fatty liver disease (NAFLD) has been associated with very high risk of right-sided cancer (13 of 199 patients with NAFLD having colonoscopy had a proximal CRC [24]). 

Clinical behaviour in the metastatic setting may also vary by initial site. Patients with proximal tumours are more likely to present with locally advanced disease, more likely to have poorly differentiated tumours [12, 20, 21], and more likely to develop peritoneal carcinomatosis (10.3% in proximal versus 6.2% in distal cancers [25]). Even accounting for these differences in presentation and the differences in epidemiology, there is evidence that outcome may differ from the more proximal tumours to those patients with distal tumours; in an analysis of nearly 54,000 patients aged over 66 from the SEER database in the USA, survival was better in patients with proximal cancers that were stage 2 at diagnosis (hazard ratio (HR) 0.92: 95% confidence intervals (CI), 0.87–0.97), but was worse in patients presenting with proximal cancers that were stage 3 at diagnosis (HR 1.12: 95% CI, 1.06–1.18 [21]) compared to similar patients with distal cancers. An analysis of 10,571 patients entered into Swedish cancer registry from 2000–2008 suggested that patients with metastatic disease arising from a proximal CRC have a slightly worse prognosis than patients with metastatic disease arising from more distal tumours (HR 1.14: 95% CI 0.98–1.34) [26].

 In summary, there is evidence that even according to an anatomical classification, CRC should not all be treated as one disease; however, whilst differences between proximal and distal cancers exist, there is no discrete anatomical cut-off [12].

## 3. Histological Classification

Most colorectal cancers are histologically classified as adenocarcinomas, which can be further stratified according to the grade of the tumour, which is related to subsequent prognosis. There were initial problems with the standardisation of grading, but now a binary classification of low- versus high- grade tumours has been adopted [27]. In addition, a number of rarer histological subtypes have been described including mucinous adenocarcinoma, adenosquamous carcinoma, signet cell carcinoma, and medullary carcinoma [28]. The prognostic impact associated with most of these subtypes is unclear. Signet cell carcinoma is thought to be associated with a poor prognosis, whilst medullary cancer is particularly associated with microsatellite instability ((MSI) see below) and so may be associated with a better prognosis. The mucinous subtype (defined as >50% of extracellular mucin within the tumour mass [28]) accounts for approximately 15% of CRC. A recent meta-analysis of 44 studies and over 200,000 patients has confirmed that this sub-type is associated with a worse prognosis (HR 1.05: 95% CI 1.02–1.08) [29]. It appears that mucinous histology (similarly to other malignancies) may predict for relative resistance to chemotherapy [30].

Additional information may be gained as to likely tumour behaviour from histological evaluation of the growth pattern of the tumour. An irregular, infiltrating pattern of growth as opposed to a smooth border has been demonstrated to be an independent adverse prognostic factor. In a recent study of 1139 CRC specimens, an infiltrative growth pattern was independently associated with a poorer survival on multivariate analysis (HR 1.78: 95% CI 1.33–2.39 [31]). In addition, the presence of tumour “budding” (microscopic clusters of up to 5 undifferentiated cancer cells just ahead of the invasive front of the tumor) is associated with higher grade tumours and poorer prognosis. It is thought this phenotype may be associated with the epithelial mesenchymal transition, thought to be important in the metastatic process. Interestingly in a small study of 43 patients with K-Ras wild-type CRC, the 7 patients with high tumour budding had no response to EGFR-targeting therapies [32].

**Figure 2:**
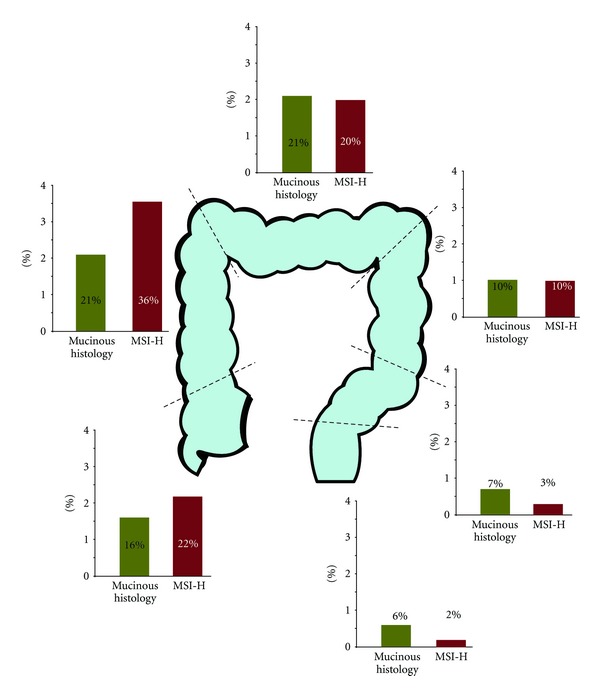
Incidence of mucinous histology and micosatellite instability by primary tumour site (data from Yamauchi et al. [12]).

In summary, histological classification and examination of other characteristics may give additional information over anatomical site in predicting prognosis and tumour response to therapy. However, there is interplay between anatomical location and histology with an increasing incidence of poorly differentiated cancers and cancers with mucinous histology in more proximal cancers [12] (See Figure 2).

## 4. Classification by Carcinogenesis Pathway

The hypothesis that there exists a molecular evolution from an adenoma to colorectal cancer following multiple oncogenic “hits” (mostly loss of tumour suppressor genes) was initially outlined by Fearon and Vogelstein in 1990 [33]. Early loss of the APC gene (mutated in familial adenomatous polyposis) is followed by later mutations including loss of the DCC gene and p53 mutation [34]. This is thought to be the mechanism of carcinogenesis in the majority of CRC, and as high rates of aneuploidy are seen is commonly referred to as the chromosomal instability (CIN) phenotype.

However, there appear to be at least 2 other important mechanisms of carcinogenesis that may be associated with different epidemiological factors and response to therapy. These include patients with hereditary nonpolyosis coli (Lynch syndrome) where there is germ-line loss of genes coding for the DNA mismatch repair (MMR) pathway, most commonly MLH1 and MSH2 [35]. Abnormalities in MMR lead to an accumulation of defects in the DNA, predominately in regions within the genome where short sequences of nucleotide bases are repeated multiple times (microsatellites) leading to multiple base changes and frame-shift mutations in these areas (the microsatellite instability- high phenotype (MSI-H)). As some of these microsatellite areas are in the promoter areas of oncogenes and tumour suppressors, this may then drive the malignant process [36, 37].

 Lynch syndrome accounts for a relatively small number of colorectal cancers (approximately 2-3%) [38]. However, a third mechanism of tumourigenesis where extensive epigenetic changes are observed, the hypermethylated phenotype (or CpG island methylator phenotype (CIMP)) of CRC [39] leads to a relatively similar MSI-H phenotype and is found in approximately 15% of CRC tumours [40]. The similarities in phenotype are probably due to the epigenetic inactivation of MLH1 and subsequent MMR dysfunction that commonly occurs in these patients. This CIMP-associated MSI-H molecular phenotype is more commonly found in patients over 70, in women, in proximal cancers (see Figure 2) and is particularly associated with B-Raf mutations (63.5% in this population versus 5% in CIN cancers and 1% in Lynch syndrome cancers [41]). 

The 2 different mechanisms of tumourigenesis (i.e., direct or epigenetic loss of MMR function) that both result in the MSI-H phenotype probably account for the bimodal distribution by age; this type of CRC is most common in patients under 50 and over 70. Differentiating MSI-H cancers from CIN cancers is important as whilst they are likely to present with a poorly differentiated cancer, matched for stage, they have a better prognosis than patients with the CIN phenotype [42–47]. An additional MSI-low phenotype related to the CIMP mechanism of tumourigenesis has been also described. This may be due to epigenetic changes leading to dysfunction in other members of the MMR pathway apart from MLH1, in particular MSH3 [48]. The clinical significance of these tumours is unknown, in particular as to whether prognosis and response to therapy should be regarded as different from CIN tumours. 

Apart from the increased proportion of MSI-H tumours found proximally, there may be an additional interplay with anatomical site (see Figure 2); it has been suggested that the relatively uncommon MSI-H tumours within the rectum (where MSH6 defects may be more common than MLH1 and MSH2) may have a different oncogenic profile [49] and may be associated with worse outcome than the more proximal MSI-H tumours and one that is similar in prognosis to that of CIN phenotype tumours [50]. 

There has been considerable controversy about whether patients with MSI-H derive benefit from 5-Flourouracil (5-FU) chemotherapy; possibly as functional MMR may be important in 5-FU activity. It may be difficult to differentiate this effect from the overall improved prognosis in patients with MSI-H tumours [47]. It was initially suggested in a review of 570 patients (95 were MSI-H) that there was a significant interaction between MSI status and efficacy of adjuvant 5-FU, with no benefit in the MSI-H group (HR 1.42: 95% CI 0.36–5.56) among patients with stage III cancer receiving 5-FU) [44]. The large confidence intervals impaired the interpretation of this analysis, but this initial finding was confirmed in a pooled analysis of 1027 patients where 5-FU seemed to be associated with a detrimental outcome in patients with stage III MSI-H cancers (HR 2.95: 95% CI 1.02–8.54 for patients receiving 5-FU) [51]. However, in an analysis of 542 patients enrolled on the National Surgical Adjuvant Breast and Bowel Project (NSABP) C01 to C04 trials, there was no interaction between MSI status and efficacy of 5-FU [46]. Similarly in the Quasar study which enrolled 1,913 patients, no interaction between MSI and efficacy of adjuvant 5-FU was seen [47]. 

Analysis of any potential impact of MSI on 5-FU sensitivity is complicated by the different demographics (associated with different comorbidities which may impact chemotherapy tolerability) and tumour characteristics that are found in patients with the MSI-H and the CIN phenotype. In addition, it should be remembered that the MSI-H tumours represent 2 different groups, the Lynch syndrome patients and those with a CIMP phenotype, and it is possible these may have a differential response to chemotherapy [42]. There has been a recent effort to specifically examine patients with the CIMP phenotype, either on demographic criteria or on assessment of methylation status of the tumour. In the largest study of 2141 tumours, the 99 patients aged under 55 with MSI-H (presumed to be Lynch syndrome) benefited from 5-FU (HR 0.31: 95% CI 0.14–0.70), but the 245 patients aged over 55 with MSI-H tumours (presumed CIMP phenotype) had no benefit (HR 1.50: 95% CI 0.82–2.74) [42]. Unfortunately, the findings of 2 studies that assessed methylation in the tumour to directly identify the CIMP phenotype contradict one another with one suggesting benefit from 5-FU chemotherapy [52] and one suggesting a lack of benefit [53]; however, the numbers of tumours with the CIMP phenotype were relatively small in both studies. 

There does not seem to be any major impact of the MSI phenotype on response to other cytotoxics such as irinotecan, oxaliplatin, or mitomycin-based chemotherapy [54–58]. It may be that in the future differentiating the Lynch syndrome MSI-H tumours from CIMP phenotype, MSI-H tumours may be important in predicting response to 5-FU and guiding choice of therapy. Trials altering adjuvant therapy on the basis of these molecular markers in CRC have been undertaken but have yet to report, for example, the ECOG-E5202 trial in which patients with resected high-risk stage 2 MSI-H tumours are observed whilst those with microsatellite stable tumours receive chemotherapy (clinical trials.gov identifier NCT00217737). 

## 5. Presence of Oncogenic Mutations

The development of monoclonal antibodies targeting the epidermal growth factor receptor led to the first major implementation of molecular profiling into the management of CRC. EGFR signals through the K-Ras oncogene which is mutated in approximately 45–50% of patients with CRC [12, 59]. It has now been conclusively demonstrated that patients with activating K-Ras mutations in the tumour do not benefit from therapy with EGFR-targeted therapy [1, 60–62]. This is presumably due to constitutive activation of the pathway that is not amenable to blockade further upstream, or possibly an effect on EGFR expression [63]. This has led to the routine use of differential treatment algorithms in patients with wild-type K-Ras from patients where an activating mutation is present [13].

However, not all patients with wild-type K-Ras benefit from EGFR therapy. This led to a search for other mutations in the EGFR signalling cascade that might predict for resistance [59, 64]. The most common abnormalities detected to date are mutations in B-Raf (which seems to be mutually exclusive with K-Ras) [12, 34, 59, 65], in the p110 alpha subunit of phosphatidylinositol 3-kinase (PI3KCa) [66–68] and the relatively rare N-Ras mutation [59, 69]. In addition, activation of alternate oncogenic growth factor receptors such as HER-3, IGFR and c-Met that can activate the survival and growth pathways downstream of K-Ras may play a role in resistance [70, 71].

Mutation in B-Raf appears to be associated with a worse prognosis in patients with CRC particularly if it arises in the context of CIN cancer pathway [65, 72–76]. The small numbers of B-Raf mutated patients and their poor prognosis have made it difficult to confirm the lack of responsiveness to EGFR inhibitors as robustly as has been demonstrated in the K-Ras mutated tumours. Most series suggest no benefit from EGFR therapies [74–76]. In a large series which identified 24 patients with B-Raf tumours having 3rd line cetuximab, 2 patients had a response [64] but this response rate was substantially lower than patients with no mutations in the EGFR pathway (8% versus 41%). There are a number of agents targeting B-Raf that are either licensed or in advanced clinical development such as vemurafenib (licensed for patients with the V600 mutation in melanoma), sorafenib, and dabrafenib. Vemurafenib may have no activity in CRC due to activation of redundant pathways [77], but sorafenib may have some activity as it has a broader inhibitory profile and affects other important processes such as angiogenesis [78].

Mutations in PI3KCa may be found in between 10–20% of CRC and can coexist with both K-Ras and B-Raf mutations [67]. Mutations in exon 9 are more common in tumours with the CIN phenotype whilst exon 20 mutations are more common in the MSI-H tumours [79]. Mutations in PI3KCa may be linked with a worse prognosis, particularly if both exons 9 and 20 are mutated (HR 2.68: 95% CI 1.24–5.77 [66]). Mutations in exon 20 rather than exon 9 may be particularly associated with resistance to EGFR-targeting therapies [64], but both mutations may be important in predicting response to novel agents targeting this protein [80].

 It now appears that the majority of CRC tumours will have at least one mutated cancer-related gene (39 of 40 tumours when 125 cancer-relevant genes were deep-sequenced), and many of these abnormalities may predict either response or resistance to therapy [34]. A number of questions remain unanswered about the impact of these oncogenic mutations on the outcome and optimal therapy for these patients. The most pressing question remains how to optimize the treatment of patients with resistance mutations. K-Ras has proved an extremely difficult target to directly drug, although downstream proteins that conduct signals from K-Ras to the nucleus, such as Mek, may be amenable to blockade [81]. 

## 6. Nononcogene Targets for MTAs

Targeting addiction to abnormal signalling of oncogenes has been one of the main thrusts of MTA development, but dependence on growth factors is only one of the hallmarks of the cancer cell [82]. Targeting angiogenesis has been shown to be a valid target in CRC with minor improvements in overall survival when bevacizumab is added to standard chemotherapy [1]. However, it appears that not all patients benefit equally. Despite many efforts, a predictive signature of likely benefit from antiangiogenic therapy has yet to be determined in CRC, but this will be important going forward in the development of these agents. 

Abnormalities in the pathways that control apoptosis may result in intrinsic or acquired resistance to therapy [83]. These abnormalities may arise either in the extrinsic apoptosis pathway which is triggered by the membrane-embedded death receptors [84, 85] or more commonly the intrinsic pathway, where the interplay of proapoptotic and antiapoptotic members of the BCL-2 family results in the release of mitochondrial contents (and subsequent apoptosis) following DNA damage, chemotherapy, or cellular stress [86–88]. A number of agents targeting these abnormalities are in advanced development [89–91], but it will be vital to determine the exact abnormalities in the apoptotic pathway in each individual cancer [92], as this will predict which of these new agents are most likely to be beneficial.

There is also an increasing recognition of the importance of evasion of the host immune response in the survival and metastasis of malignant cells. MSI-H tumours are associated with a more prominent lymphocytic response within the tumour [40], and this might partially account for improved survival in patients with these cancers. Manipulation of the host response may be less liable to mutation and therapeutic escape than directly targeting the cancer cell. Agents targeting the immune response are now available, for example, ipilimumab a monoclonal antibody that blocks the action of CTLA4 and decreases immune tolerance is licensed in melanoma, and may now be evaluated in other solid tumours [93]. Blocking the proinflammatory local tumour environment may be as important in anticancer therapy, in particular, in preventing metastases [82]. This was recently shown in the a subset of patients with CRC enrolled on trials examining the effect of aspirin in the prevention of vascular events [94] where less metastases were seen on the intervention arm. However, it still remains to determine the host and tumour characteristics that will predict the most benefit from manipulation of the immune system. 

In summary, K-Ras mutation status is already used to subclassify CRC tumours [13]. As the number of MTAs available for routine clinical use increases, molecular profiling (both of oncogenic growth factors and of other proteins involved in the maintenance and spread of the malignant cells) to identify patients most likely to benefit from these novel therapies is going to become increasingly important.

## 7. Interplay of Host Factors

**Figure 3:**
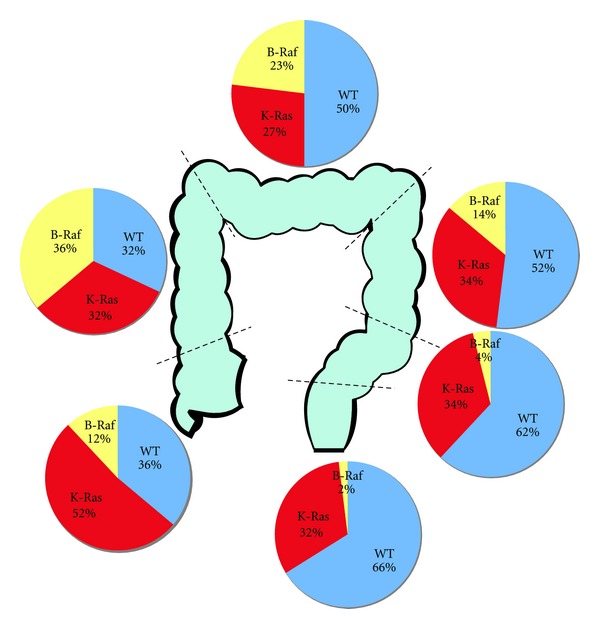
Incidence of K-Ras and B-Raf mutations by primary tumour site (data from Yamauchi et al. [12]).

The importance of the host interaction with tumour characteristics should not be underplayed in any discussion about the potential classification of CRC. We have already discussed the substantial interplay of patient age and gender sex with the anatomical position and molecular classification of CRC (see Figures 1, 2, and 3). Much of the work to date in classification of CRC has been performed in Caucasian and to a lesser extent Asian populations. There is some evidence of a differential impact of tumour factors in different ethnic populations, for example Afro-Americans have a higher rate of proximal tumours with an increased frequency of K-Ras mutations but a similar rate of MSI-H tumours [95]. In patients with CIN tumours, prognosis is much worse than in Caucasians, whilst in patients with MSI-H tumours, it is equivalent; the exact cause of this difference is at present unclear [95]. In addition, host factors such as drug metabolism and function of the immune system may affect both the efficacy and tolerability of therapy, which may as much as an effect on eventual outcome of therapy as individual tumour factors.

## 8. Problems Arising from Classifying CRC

There are a number of problems that are arising as we seek to classify patients' tumours further. In order to give additional information which can be used to guide patient care, there need to be standardised validated assays that have a low assay failure rate and give results in a timely manner. In addition, a relatively large amount of material that has been examined by a histopathologist and been shown to have a high proportion of tumour cells may need to be available to allow molecular classification. 

A number of ethical issues may also arise, in particular in the classification of the tumour by tumorigenic pathway. We have discussed that determining the patients with MSI-H tumours is important in terms of both prognosis and guiding therapy. A number of these patients will have Lynch syndrome; the diagnosis of which will have an impact on both them and their families. This additional information can give additional distress at the already stressful time of a new cancer diagnosis [96] and raises the question as to when and how patients are consented to have their tumour assessed for the presence of MSI.

One of the major difficulties that is now being encountered in developing personalised treatment strategies is the heterogeneity of molecular abnormalities within the tumour of an individual patient [97–99]. It was initially thought that as cancer is a clonal disease, and that many of the abnormalities targeted by MTAs drive the oncogenic process, that these abnormalities would be conserved throughout the tumour and in metastases. There is increasing evidence that this hypothesis is not true, and there may be discordance in the mutation profile and expression of important oncogenes such as K-Ras and PIK3CA [99, 100]. Selection pressure of therapy may exacerbate the observed heterogeneity [97]. The exact impact of this phenomenon on treatment is still to be evaluated; it may be that monitoring the molecular profile of circulating tumour cells will allow early detection of evolving resistance mechanisms to guide changes in therapy [101].

## 9. Adapting Trial Design to Take Account of Disease Heterogeneity

The increasing stratification of cancer and the development of personalised treatment strategies require an examination of how clinical trials are designed in the era of the development of MTAs [102]. Failure to account for disease heterogeneity may lead to the abandonment of an effective treatment in a particular subpopulation (an example of this may be the development of trastuzumab, which only has activity in patients with breast and gastric cancer that over-express the target protein). 

The gold standard method to examine the potential of a predictive biomarker is a randomised clinical trial (with mandatory provision of biopsy material for biomarker analysis) and a preplanned analysis of the impact of biomarker expression on treatment outcome. Although the initial assessment of K-Ras status as a predictive biomarker of resistance to EGFR targeting antibodies was performed in a retrospective manner [60], similar findings have been confirmed in prospective studies [62]. This randomised approach may be vital if a biomarker is associated with both a prognostic impact and predicts response to therapy (as a case in point B-Raf mutation is both associated with a worse prognosis and may predict resistance to EGFR targeting therapies, as discussed above).

However, there are a number of problems with this approach. The addition of MTAs to therapy may actually be detrimental in a setting where it does not add anything to efficacy [103, 104]. This is probably because of problems delivering full doses of chemotherapy in combination with MTAs in some patients due to overlap of the toxicity profiles or intolerability [105, 106]. This suggests where possible trials evaluating MTAs should be restricted to patients most likely to benefit. This can be difficult to determine upfront; for example, for a long time, it was not certain what (if any) biomarker would predict for sensitivity to EGFR therapy, and as discussed earlier, it is still uncertain which patient group will derive most benefit from antiangiogenic therapy. In addition, it was relatively easy to evaluate the impact of K-Ras mutation as this is expressed in approximately 50% of patients (see Figure 3) and so the numbers of patients expressing the putative biomarker and those with wild-type K-Ras were relatively balanced, allowing a well-powered retrospective statistical analysis. However, as many of the MTAs will only be effective in a relatively small proportion of the population, it may be difficult to complete a statistically powered trial in that population. The best method to evaluate MTAs in small populations may be large multicentre trials that collaborate to stratify patients to the therapy from which they are most likely to benefit.

As we personalise medicine and look to treat the increasing heterogeneity of cancer, an ideal format might be considered to be the so called “*N* = 1” trial where patients act as their own control [107, 108]. This approach has been advocated in a number of nonmalignant settings. A simulation of this approach was attempted in a trial of individualised therapy in patients with a range of tumour types, where the progression-free survival on an individualised treatment regimen (advised on target expression in the tumour) was compared to progression-free survival on their previous regimen [109]. Unfortunately the trial set-up did lay it open to potential systemic biases [110], but the approach is of interest. 

## 10. Conclusions

In patients with localised disease rectal cancer will continue to be treated as a separate group due to the differences in surgical approach and the evidence for the benefit of chemo-radiotherapy. In patients with metastatic disease, there is no evidence in terms of epidemiology, histology, and molecular profiles to suggest clear differences between the behaviour of tumours arising within the colon and rectum or the midgut and hindgut. Instead, there is a complex interplay of anatomical, histological, chromosomal, and molecular factors that suggest there is a spectrum of different diseases that are presently labelled colorectal cancer [12]. At one of end of the spectrum are distal cancers with a CIN phenotype, whilst more proximally MSI-H cancers, cancers with mucinous histology and tumours with activating B-Raf mutations are more likely to be found (see Figures 2 and 3). There are marked differences in tumour prognosis and response to therapy between these 2 ends of the spectrum, and these differences are likely to increase in the future with increasing integration of MTAs into therapy. Classification according to molecular and pathological factors evaluation is needed [6] and will need to continue to adapt to identify patients most likely to benefit from novel therapeutics. Innovative trial designs and multicentre collaborations will be required to provide the evidence base that will enable clinicians to determine which combinations of therapy are optimal for an individual patient's cancer.
